# Rhabdomyolysis as Potential Late Complication Associated with COVID-19

**DOI:** 10.3201/eid2607.200445

**Published:** 2020-07

**Authors:** Min Jin, Qiaoxia Tong

**Affiliations:** Cancer Center, Union Hospital, Tongji Medical College, Huazhong University of Science and Technology, Wuhan, China (M. Jin);; Department of Infectious Diseases, Union Hospital, Tongji Medical College, Huazhong University of Science and Technology, Wuhan (Q. Tong)

**Keywords:** severe acute respiratory syndrome coronavirus 2, SARS-CoV-2, rhabdomyolysis, COVID-19, 2019 novel coronavirus disease, viruses, respiratory diseases, zoonoses, Wuhan, China

## Abstract

We describe a patient in Wuhan, China, with severe acute respiratory syndrome coronavirus 2 infection who had progressive pulmonary lesions and rhabdomyolysis with manifestations of lower limb pain and fatigue. Rapid clinical recognition of rhabdomyolysis symptoms in patients with severe acute respiratory syndrome coronavirus 2 infection can be lifesaving.

Recently, the outbreak of severe acute respiratory syndrome coronavirus 2 (SARS-CoV-2) infection in Wuhan, China, has attracted great attention worldwide (*1*). SARS-CoV-2, the cause of 2019 novel coronavirus disease (COVID-19), belongs to the β-coronavirus family, which also includes 2 other highly pathogenic human coronaviruses (*2*): severe acute respiratory syndrome coronavirus and Middle East respiratory syndrome coronavirus. Fever, cough, myalgia, and fatigue are the common symptoms of COVID-19, whereas expectoration, headache, hemoptysis, and diarrhea are relatively rare (*3*).

Rhabdomyolysis is a life-threatening disorder that manifests with myalgia, fatigue, and pigmenturia; it can also manifest as acute renal failure (*4*). The inducing factors of rhabdomyolysis include autoimmune myopathies, septicemia, electrolyte abnormalities, substance abuse, alcohol use, or infection (*5*). Viral infection, especially influenza virus infection, can lead to rhabdomyolysis (*6*). We report rhabdomyolysis related to COVID-19 in Wuhan, China.

A 60-year-old man in Wuhan sought care in February 2020 for a 6-day history of fever up to 38.3°C and cough. Chest computed tomography performed 3 days before in another hospital showed that the texture of both lungs was thickened and scattered with ground glass shadows (Figure). When the patient arrived, he was alert; heart rate was 89 bpm, blood pressure was 135/91 mm Hg, respiratory rate was 18 breaths/min, temperature was 38.5°C, and saturation of peripheral oxygen was 93%. Physical examination revealed a rough breath sound in the lungs. Laboratory findings included mild leukopenia (3.31 × 10^9^ neutrophils/L [reference 3.5–9.5 × 10^9^ neutrophils/L]), increased lactate dehydrogenase (280 U/L [reference 109–245 U/L]), and increased C-reactive protein (111 mg/L [reference 0–8 mg/L]) (Table). Results were in the normal range for creatine kinase (CK) and indicators of hepatic and kidney function. Screenings for common infectious diseases were negative. Real-time reverse-transcription PCR analysis of the patient’s throat swab specimen indicated SARS-CoV-2 infection. 

**Figure:**
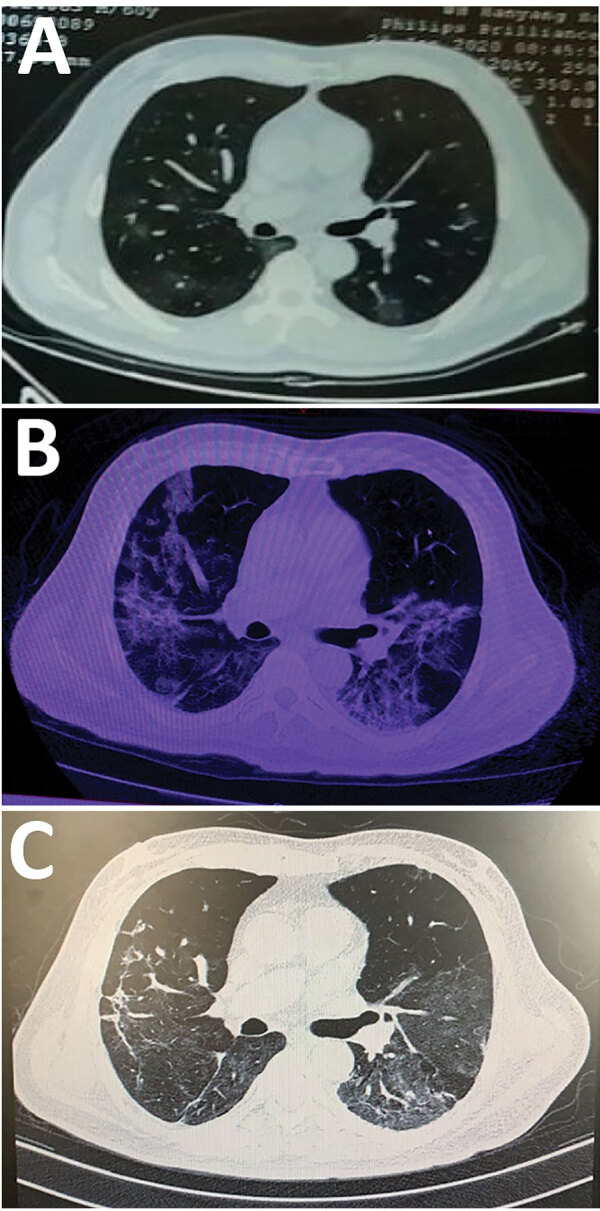
Computed tomography (CT) scan of the lungs of a 60-year-old man before and after severe acute respiratory syndrome coronavirus 2 (SARS-CoV-2) infection and rhabdomyolysis, Wuhan, China, 2020. A) CT scan before diagnosis of SARS-CoV-2 infection (3 days before hospital admission) revealed the lungs were thickened and scattered with ground-glass shadows. B) CT scan after diagnosis of SARS-CoV-2 infection with rhabdomyolysis (on hospital day 10) indicated that most of both lungs were covered with ground-glass shadows. C) CT scan after SARS-CoV-2 infection with rhabdomyolysis (on hospital day 19) indicated that pulmonary inflammation was improved.

**Table:** Biochemistry and blood gas parameters of a 60-year-old man with severe acute respiratory syndrome coronavirus 2 infection and rhabdomyolysis, by day of hospitalization, Wuhan, China, 2020*

Parameter (reference range)	Day 1	Day 3	Day 6	Day 9†	Day 10	Day 11	Day 12‡	Day 15	Day 17	Day 20
Myoglobin (0–140 μg/L)	ND	ND	ND	>12,000	12,550	7,905	3,280	928	152	86
Creatine kinase (38–174 U/L)	47	ND	ND	11,842	17,434	14,318	11,067	2,954	1,447	251
LDH (109–245 U/L)	280	ND	ND	2,347	2,137	1,979	1,754	1,265	923	597
α-hbdh (72–182 U/L)	277	ND	ND	1,612	1,436	1,171	1,143	1,037	911	189
Amyloid A (0–10 mg/L)	746	ND	ND	429	192	105	126	93	84	25
CRP (0–8 mg/L)	111	123	206	58	45	23.4	23.4	21.4	6.1	15
ALT (5–40 U/L)	37	82	61	111	162	171	172	142	133	56
AST (8–40 U/L)	48	88	35	213	373	348	320	183	135	38
Albumin (35–55 g/L)	33.7	31.8	27.6	32.3	28.5	30.3	30	30.7	29.3	30.3
Creatine (44–133 μmol/L)	72.5	74.4	72.6	65.2	68.9	68.8	59.2	68	65.7	67.3
PH (7.35–7.45)	ND	ND	ND	ND	7.51	7.4	7.48	7.45	ND	7.40
PCO_2_ (35–45 mm Hg)	ND	ND	ND	ND	29.2	34.8	34.6	36	ND	38
PO_2_ (83–103 mm Hg)	ND	ND	ND	ND	49	147	142	120	ND	102

*α-hbdh, α-hydroxybutyrate dehydrogenase; ALT, alanine aminotransferase; AST, aspartate aminotransferase; LDH, lactate dehydrogenase; ND, not done. †Rhabdomyolysis symptoms appeared on hospital day 9. ‡Real-time reverse transcription PCR conducted on hospital day 12 was negative for severe acute respiratory syndrome coronavirus 2.

We treated the patient with oxygen inhalation, opinavir, moxifloxacin, interferon nebulization, an antitussive, and nutritional support. On day 6 after admission, the patient still had an intermittent fever up to 38°C. We broadened the antibiotic treatment to include meropenem and added methylprednisolone. His fever abated on hospital day 7. However, serologic examination showed that C-reactive protein had increased to 206 mg/L.

On hospital day 9, the patient felt pain and weakness in his lower limbs. He denied medication exposure, illicit drug use, or alcohol use. Physical examination indicated tenderness in the lower limbs. Urgent laboratory examination indicated that myoglobin was >12,000.0 μg/L (reference 0–140 μg/L), CK was 11,842 U/L (reference 38–174 U/L), lactate dehydrogenase was 2,347 U/L (reference 109–245 U/L), alanine aminotransferase was 111 U/L (reference 5–40 U/L), and aspartate aminotransferase was 213 U/L (reference 8–40 U/L) (Table). The patient’s kidney function and electrolytes were normal. Urine analysis revealed light yellow color of urine, occult blood was positive, and urine protein was suspiciously positive. These results indicated the onset of rhabdomyolysis.

In addition to the ongoing treatments, the patient was immediately treated with hydration, alkalization, plasma transfusion, gamma globulin, and symptomatic supportive therapy. On hospital day 10, the laboratory index continuously increased (Table). Blood gas analysis showed that PCO_2_ was 29.2 mm Hg (reference 35–45 mm Hg), PO_2_ was 49 mm Hg (reference 83–103 mm Hg), and pH was 7.51 (reference 7.35–7.45). A computed tomography reexamination of the lungs showed that the pulmonary lesions had substantially deteriorated (Figure). We continued the aggressive fluid therapy and maintained the acid–base balance while also continuing treatment with opinavir and moxifloxacin.

The patient reported less pain and fatigue in his lower limbs in the following days. Biochemistry and blood gas indicators gradually returned to normal levels (Table). Moreover, a second real-time reverse transcription PCR test conducted on hospital day 12 was negative for SARS-CoV-2. The patient’s symptoms improved daily, and he was again able to move his lower limbs freely.

The initial manifestations of SARS-CoV-2 infection in this patient were fever and cough. After a short period of antimicrobial drug treatment, his fever abated, but the condition of both lungs was deteriorating. Meanwhile, symptoms of rhabdomyolysis began.

General muscle pain and fatigue are common symptoms of COVID-19, but clinicians should consider the diagnosis of rhabdomyolysis when patients have focal muscle pain and fatigue (*7*). CK and myoglobin levels are important indexes for rhabdomyolysis (*5*); however, they are not tested routinely, so rhabdomyolysis is easily misdiagnosed. The key to avoid acute renal failure from rhabdomyolysis is early detection and treatment with aggressive hydration (*7*). 

We generally know very little of the multifaceted biologic characteristics of COVID-19. Moreover, to our knowledge, COVID-19–associated rhabdomyolysis has not been previously reported; therefore, clinicians might have low clinical suspicion for rhabdomyolysis.

The case we describe lacks a final etiology for rhabdomyolysis. Also, our findings are limited by the absence of virus sequencing and confirmation of rhabdomyolysis pathology analysis. However, our findings indicate that rapid clinical recognition and positive hydration treatment of COVID-19–associated rhabdomyolysis can reduce the risk for serious outcomes.
